# Ketogenic and Low-Carbohydrate Diets in Prostate Cancer: Metabolic Rationale, Preclinical Evidence, and Preliminary Clinical Data

**DOI:** 10.3390/jcm15103946

**Published:** 2026-05-20

**Authors:** Silvia Manfrini, Andrea Malgeri, Carmine Mone, Ludovica Di Francesco, Giulia Pecora, Rossella Mazzilli, Giuseppe Defeudis, Manon Yeganeh Khazrai, Antongiulio Faggiano

**Affiliations:** 1 Fondazione Policlinico Universitario Campus Bio-Medico, 00128 Roma, Italy; s.manfrini@policlinicocampus.it; 2 Area Oncologia, Università Campus Bio-Medico di Roma, 00128 Roma, Italy; a.malgeri@policlinicocampus.it (A.M.); c.mone@policlinicocampus.it (C.M.); 3 Scienza dell’Alimentazione e della Nutrizione Umana, Università Campus Bio-Medico di Roma, 00128 Roma, Italy; l.difrancesco@policlinicocampus.it (L.D.F.); m.khazrai@policlinicocampus.it (M.Y.K.); 4 Unità di Endocrinologia, Diabetologia e Andrologia, Dipartimento di Medicina Clinica e Molecolare, AOU Sant’Andrea, Università Sapienza di Roma, 00185 Roma, Italy; g.pecora@uniroma1.it (G.P.); rossella.mazzilli@uniroma1.it (R.M.); antongiulio.faggiano@uniroma1.it (A.F.); 5 Department of Theoretical and Applied Sciences, eCampus University, 22060 Novedrate, Italy

**Keywords:** prostate cancer, ketogenic diet, androgens, low-carbohydrate diets, dietary patterns

## Abstract

**Background:** Prostate cancer (PCa) is the most commonly diagnosed malignancy in men and a leading cause of cancer-related mortality worldwide. Growing evidence indicates that metabolic syndrome components, including obesity, insulin resistance, and hyperglycemia, contribute to PCa development, and progression to more aggressive form. At the same time, standard treatments such as androgen deprivation therapy (ADT) and androgen receptor pathway inhibitors (ARPIs) significantly improve oncologic outcomes but are associated with adverse metabolic effects, including increased fat mass, insulin resistance, and sarcopenia, potentially worsening patients’ overall metabolic profile and quality of life. Tumor progression in PCa is strongly driven by androgen receptor (AR) signaling, which is closely linked to cellular metabolic reprogramming, highlighting metabolism as a potential therapeutic target. **Aim**: The aim of this study was to evaluate and synthesize current evidence on the role of the ketogenic diet (KD) in PCa, with particular emphasis on its interaction with hormonal therapies, underlying metabolic and endocrine mechanisms, and its potential application as an adjunctive strategy in integrated oncologic care. **Results**: The KD, characterized by high fat and very low carbohydrate intake, induces a metabolic state of ketosis that reduces circulating glucose, insulin, and insulin-like growth factor 1 (IGF-1), potentially counteracting metabolic alterations associated with PCa and its treatments. Preclinical studies consistently demonstrate that carbohydrate restriction and KD can slow tumor growth, modulate key oncogenic pathways such as PI3K/AKT/mTOR, reduce systemic insulin signaling, and enhance survival in prostate cancer models. Additionally, emerging evidence suggests possible synergistic effects when KD is combined with standard therapies, including ADT and immunotherapy. Clinical data, although limited, indicate that low-carbohydrate dietary interventions may improve metabolic parameters and could delay biochemical progression, as suggested by increased prostate-specific antigen (PSA) doubling time. However, results across studies remain heterogeneous, and robust evidence on long-term oncologic outcomes is lacking. **Conclusions**: Overall, the KD represents a promising but still experimental strategy in PCa management, requiring careful nutritional supervision to avoid adverse effects such as unintended weight loss or sarcopenia. Further well-designed randomized clinical trials are needed to clarify its safety, efficacy, and role in routine clinical practice.

## 1. Introduction

Prostate cancer (PCa) remains the most commonly diagnosed malignancy among men in developed countries and a leading cause of cancer-related mortality [1]. Multiple epidemiological studies have shown that metabolic syndrome traits—including abdominal obesity, hypertension, type 2 diabetes, and insulin resistance—are linked to worse PCa outcomes. In particular, elevated body mass index (BMI) has been associated with higher PCa mortality and progression to more aggressive forms. The EPICAP study also highlighted central adiposity as an independent risk factor for PCa [2,3,4,5,6].

PCa carcinogenesis is critically dependent on androgen receptor (AR) signaling. In fact, the AR remains a central oncogenic driver, promoting tumor growth, survival, and progression even in castration-resistant settings [7,8]. Importantly, AR activity is also intricately linked to cellular metabolism [9]. This includes increased lipid synthesis, altered glucose utilization, and enhanced mitochondrial function, creating a unique metabolic phenotype within the tumor microenvironment [10]. While AR remains a dominant force in shaping PCa metabolism, additional regulators—such as insulin, insulin-like growth factor 1 (IGF-1), inflammatory cytokines, and dietary substrates—also contribute to this complex landscape, offering novel opportunities for metabolic-targeted therapies.

In this context, androgen deprivation therapy (ADT) has long served as the cornerstone of treatment for advanced PCa [11,12]. More recently, androgen receptor pathway inhibitors (ARPIs) such as enzalutamide, apalutamide, and darolutamide have further improved outcomes by providing deeper AR blockade in both hormone-sensitive and castration-resistant disease [13,14,15,16,17,18]. Despite their efficacy, these therapies are associated with a range of adverse effects, including fatigue, cognitive impairment, cardiovascular risk, and profound metabolic disturbances such as increased fat mass, insulin resistance, and sarcopenia [19,20,21].

Therefore, these findings point toward a potential role for nutritional and metabolic factors in PCa pathogenesis and progression.

These therapy-induced changes closely mirror the features of metabolic syndrome and highlight the potential for nutritional interventions to mitigate side effects or enhance therapeutic response.

Among these, the ketogenic diet (KD)—a high-fat, low-carbohydrate dietary pattern that induces a metabolic shift toward ketone body utilization—has emerged as a promising strategy. By lowering circulating glucose, insulin, and IGF-1 levels, the KD may counteract some of the endocrine and metabolic consequences of ADT and ARPI, while simultaneously exploiting cancer-specific vulnerabilities in glucose and lipid metabolism [22,23,24].

This review aims to synthesize current evidence on the role of the KD in PCa, focusing on its interaction with hormonal therapies, underlying metabolic and endocrine mechanisms, and its potential as an adjunctive strategy in integrated oncologic care.

## 2. Methods

### 2.1. Review Design

This study was conducted as a narrative review aimed at providing a comprehensive and up-to-date overview of the literature on the role of the KD in PCa.

### 2.2. Data Sources and Search Strategy

A literature search was performed in major electronic databases, including PubMed/MEDLINE, Scopus, and Web of Science, up to July 2025. The search strategy combined relevant keywords and Medical Subject Headings (MeSH) related to KD in PCa. Additional articles were identified through manual screening of reference lists of relevant papers.

### 2.3. Eligibility Criteria

Studies were considered eligible if they were published in English, focused on the KD in PCa, and provided relevant data or conceptual insights aligned with the objectives of this review. Both original research articles and relevant reviews were considered. Studies were excluded if they were not pertinent to the scope of the review or lacked sufficient methodological or conceptual contribution.

### 2.4. Study Selection

Articles were screened based on title and abstract, followed by full-text evaluation when necessary. Selection was guided by relevance to the topic and the aim of providing a balanced and informative synthesis of the available evidence.

### 2.5. Data Extraction and Synthesis

Key information from the included studies was extracted, including study characteristics, main findings, and relevant methodological aspects. Data were synthesized qualitatively using a narrative approach, with the aim of identifying key themes, areas of agreement or controversy, and gaps in the current literature.

## 3. Ketogenic Diet

The KD refers to any nutritional strategy designed to induce a state of ketosis, achieved through carbohydrate restriction, typically to less than 30–50 g per day. After a few days of carbohydrate restriction, the body compensates by mobilizing free fatty acids from adipose tissue, which are then converted into ketone bodies to serve as an alternative energy source [25,26,27].

Since various definitions and classifications of KDs exist in the literature, it is important to distinguish them clearly. The Very Low-Calorie Ketogenic Diet (VLCKD), Low-Calorie Ketogenic Diet (LCKD), and Isocaloric Ketogenic Diet (ICKD) primarily differ in their total caloric content: the VLCKD typically provides 700–800 kcal/day, the LCKD provides between 700–800 kcal and up to the total energy expenditure, while the ICKD matches the individual’s total energy expenditure. Regarding macronutrient composition, all KDs must maintain protein intake within a range of 0.8–1.5 g of protein per kilogram of ideal body weight per day to preserve ketosis, since excess protein under caloric restriction can be converted to glucose via gluconeogenesis. In terms of fat intake, a VLCKD usually provides up to 30–40 g of fat daily (often limited to 10–20 g/day), an LCKD typically exceeds 30–40 g of fat per day, whereas an ICKD derives approximately 70–80% of total caloric intake from fat [25].

In 2024, the Italian Society of Nutraceuticals and the Italian Association of Dietetics and Clinical Nutrition introduced updated terminology for the VLCKD, now called Very-Low-Energy Ketogenic Therapy (VLEKT). VLEKT is defined by a very low energy intake (650–800 kcal/day), minimal carbohydrates (<30 g daily from vegetables), and limited fat (only about 20 g per day, usually from extra virgin olive oil). A minimum intake of 2 L of water per day, along with mandatory supplementation of vitamins, electrolytes, and omega-3 polyunsaturated fatty acids, is required. According to the scientific literature, VLEKT can be safely followed for up to 12 weeks, after which a gradual reintroduction of carbohydrates is recommended [26]. The classic KD is defined by a fat-to-combined-carbohydrate-and-protein ratio of 4:1, providing approximately 90% of total daily calories from fat. However, due to its restrictive nature, patient adherence has historically been low [27]. In recent years, more flexible ketogenic protocols have been developed to improve adherence. Modern clinical KDs typically supply at least 80% of total calories from fat, with fat-to-carb-plus-protein ratios of 2:1 or 3:1. VLEKT is also an established protocol used for different clinical goals. Alternative approaches include the medium-chain triglyceride (MCT) KD and the modified Atkins diet [28].

Originally, the KD was introduced in the early 20th century as a treatment for drug-resistant epilepsy, but its potential benefits for weight loss and other metabolic conditions have been widely explored only in recent decades.

In 2019, Caprio et al. published clinical guidelines supporting the use of the very-low-calorie KD as a therapeutic tool for managing obesity and obesity-related comorbidities [29]. More recently, in 2024, the Italian Association of Medical Oncology (AIOM) recommended that, for patients receiving hormonal therapies—such as for breast or PCa—caloric restriction under specialist supervision may be indicated to prevent or treat overweight and obesity [30].

The specific type of KD should be selected according to the clinical context and therapeutic goal. For cancer patients, the protocols most frequently studied include the classic 2:1 isocaloric KD or the MCT KD. In some cases, researchers have tested a 900 kcal KD or a KD with a 20% caloric restriction relative to total daily energy expenditure. A 2:1 ketogenic ratio is generally sufficient for oncology settings, whereas the stricter 4:1 ratio commonly used for refractory epilepsy may not be necessary [31].

## 4. Clinical Effects of Ketogenic Diet on Other Tumors

KDs may exert their effects through multiple interconnected metabolic and molecular mechanisms. By markedly reducing carbohydrate intake, KDs lower circulating glucose, insulin, and IGF-1 levels, thereby attenuating activation of the PI3K/AKT/mTOR signaling pathway, which plays a central role in tumor growth and proliferation. In parallel, the metabolic shift toward fatty acid oxidation and ketone body production creates a bioenergetic environment that may be less favorable for cancer cells, particularly those with limited metabolic flexibility or mitochondrial dysfunction.

Ketone bodies, especially β-hydroxybutyrate (BHB), also act as signaling molecules. BHB has been shown to inhibit class I histone deacetylases (HDACs), thereby modulating gene expression, promoting oxidative stress resistance in normal cells, and potentially impairing tumor cell proliferation. In addition, ketogenic diets may influence the tumor microenvironment by reducing systemic inflammation, decreasing pro-inflammatory cytokines, and modulating immune cell function, including enhancement of cytotoxic T-cell activity.

Furthermore, reduced glucose availability may impose metabolic stress on tumor cells that rely heavily on aerobic glycolysis (the Warburg effect), limiting their ability to sustain rapid growth. At the same time, normal cells are better able to adapt to ketone utilization, potentially improving the therapeutic window.

Collectively, these mechanisms provide a strong biological rationale for the potential anticancer effects of ketogenic dietary interventions [32,33,34,35].

KDs have been investigated across a variety of tumor types, including glioblastoma, breast, pancreatic, lung, ovarian, and colorectal cancers, with heterogeneous but overall exploratory clinical results [36,37,38,39,40,41,42,43,44,45,46,47,48,49,50,51,52,53,54]. Rather than consistent effects on tumor regression or survival, most studies report improvements in metabolic parameters, body composition, quality of life, and, in some cases, treatment tolerability.

For example, early clinical observations in glioblastoma patients suggested potential benefits in terms of metabolic control and disease stabilization [36,37,38,39,40,41], although results were inconsistent across studies and often limited by small sample size and variable adherence to dietary protocols [42]. Similarly, studies in other tumor types, including breast and gynecological cancers, have reported improvements in body composition, fatigue, and metabolic biomarkers, but without consistent evidence of survival benefit [43,44,45,46,47,48]. In patients undergoing radiotherapy or chemotherapy, ketogenic interventions have been associated in some cases with improved metabolic profiles and reduced treatment-related side effects [49,50,51].

However, two recent meta-analyses including cancer patients across multiple tumor types did not demonstrate significant effects of ketogenic diets on fasting glucose, insulin levels, lipid profile, or body weight [52,53,54]. Overall, the heterogeneity of study designs, tumor types, and dietary protocols, together with limited adherence, significantly complicates interpretation of the available evidence.

Importantly, these findings cannot be directly extrapolated to prostate cancer, which exhibits distinct metabolic features, including androgen receptor-driven metabolic reprogramming and a higher reliance on lipid metabolism compared with many other solid tumors. Nevertheless, the shared involvement of key pathways such as insulin/IGF-1 signaling, inflammation, and tumor bioenergetics supports the biological plausibility of ketogenic interventions as a systemic metabolic strategy.

## 5. Prostate Cancer Metabolism

PCa displays a distinctive metabolic phenotype that differentiates it from many other solid tumors. Unlike most malignancies that predominantly depend on aerobic glycolysis (the Warburg effect), normal prostate epithelial cells accumulate and secrete citrate due to high intracellular zinc levels that inhibit mitochondrial aconitase, resulting in truncated citrate oxidation. During malignant progression, intracellular zinc concentrations decline, restoring aconitase activity, enabling complete citrate oxidation, and enhancing flux through the tricarboxylic acid (TCA) cycle and oxidative phosphorylation to sustain tumor growth and survival [55,56,57].

A central driver of this metabolic rewiring is the androgen receptor (AR), which functions as a key nuclear receptor regulating prostate development and homeostasis. The AR mediates androgen signaling and orchestrates the expression of genes involved in proliferation, differentiation, and metabolic adaptation. By reprogramming cellular energy metabolism, AR establishes a unique metabolic landscape that has been recognized for over a century and remains fundamental to PCa pathophysiology. Consequently, inhibition of AR signaling represents the mainstay of therapy for advanced disease [58].

Recent evidence highlights how metabolic interventions may synergize with conventional therapies. For instance, the KD, by promoting ketone body production and restricting carbohydrate intake, may reduce glucose availability for highly glycolytic cancer cells while supplying alternative energy substrates for normal tissues [24]. Additionally, ADT profoundly alters PCa metabolism, increasing lipid and amino acid utilization, which may contribute to therapy resistance and disease progression [59,60]. Understanding these metabolic dependencies provides a rationale for integrating dietary or metabolic strategies, such as ketogenic interventions, as adjuvant approaches to modulate tumor growth and potentially enhance treatment efficacy.

Specifically, in hormone-sensitive prostate cancer, tumor growth remains largely driven by AR signaling and its associated metabolic programs, including lipid synthesis and glucose utilization [8,55]. In this context, KDs may exert indirect effects primarily through systemic metabolic modulation, including reductions in insulin and IGF-1 signaling pathways converging on PI3K/AKT/mTOR signaling.

In castration-resistant prostate cancer (CRPC), disease progression is accompanied by metabolic reprogramming, with increased reliance on lipid metabolism and mitochondrial oxidative phosphorylation, reflecting enhanced metabolic plasticity [12]. This metabolic shift may alter sensitivity to glucose restriction and suggests context-dependent effects of dietary interventions.

A distinct entity is neuroendocrine prostate cancer (NEPC), characterized by loss of AR dependence and aggressive clinical behavior [12]. However, specific evidence regarding KD interventions in this subtype is currently unavailable.

Overall, while KD interventions may differentially interact with PCa subtypes based on underlying metabolic dependencies, current evidence is insufficient to draw definitive conclusions.

## 6. Effects of Different Dietary Patterns on Prostate Cancer Progression

Several studies have shown inverse associations between higher diet quality and PCa risk, but evidence on post-diagnosis dietary patterns and outcomes remains limited.

In the Physicians’ Health Study, Yang et al. reported that a Western dietary pattern (high in red meat, high-fat dairy, refined grains) was linked to a 2.5-fold increase in PCa-specific mortality (HR = 2.53; 95% CI 1.00–6.42), while a prudent pattern (rich in vegetables, fruits, fish, legumes, vegetable oils, whole grains) was associated with a 36% reduction in overall mortality (RR = 0.64; 95% CI 0.44–0.93) [61].

Another study by Yang et al. found that men consuming ≥3 servings/day of dairy had higher overall mortality (HR = 1.76; 95% CI 1.21–2.55), with both high-fat (HR = 1.22; 95% CI 1.08–1.38) and low-fat dairy (HR = 1.17; 95% CI 1.05–1.29) contributing to this association [62].

In the Health Professionals Follow-up Study, among 4538 non-metastatic PCa survivors, adherence to a Mediterranean diet post-diagnosis was associated with a 22% lower risk of all-cause mortality (HR = 0.78; 95% CI 0.67–0.90), though no association with PCa outcomes was found [63].

The prospective Canary Prostate Active Surveillance Study (PASS) assessed whether better diet quality (Healthy Eating Index (HEI-2015), the Alternative Mediterranean Diet (aMED), and the Dietary Approaches to Stop Hypertension (DASH) dietary patterns) post-diagnosis was linked to reduced grade progression. The HEI was developed by the U.S. Department of Agriculture (USDA) and the National Cancer Institute to evaluate adherence to the 2015 Dietary Guidelines for Americans, which emphasize foods that promote overall health, including fruits, vegetables, whole grains, and lean proteins. The aMED score reflects adherence to a Mediterranean diet, which is high in monounsaturated fats, plant-based proteins, whole grains, and fish; moderate in alcohol; and low in red meat, refined grains, and sweets. The DASH score is based on a higher intake of foods such as fruits, vegetables, whole grains, low-fat dairy, nuts, seeds, vegetable oils and legumes, alongside a reduced intake of refined grains, red and processed meats, and sodium. No significant associations were found, although these diets are generally associated with better overall health [64].

Finally, a 2024 cohort study involving 2062 men with non-metastatic PCa showed that greater adherence to a plant-based diet (with a higher consumption of vegetables, fruits, vegetable oils, whole grains, nuts and pulses) was linked to a 47% lower risk of progression (HR = 0.53; 95% CI 0.37–0.74; *p*-trend = 0.003) [65].

In conclusion, while evidence remains inconclusive due to the limited number of studies, diets rich in plant-based foods, whole grains, and anti-inflammatory components may improve PCa outcomes and merit further research.

Overall, although the available evidence remains limited and partly inconsistent, several common patterns emerge across studies. Dietary patterns characterized by high intake of red and processed meats, refined carbohydrates, and high-fat dairy products, typical of a Western diet, appear to be associated with worse prostate cancer outcomes, including increased overall and disease-specific mortality [61,62]. These associations may be mediated by increased insulin resistance, higher circulating levels of insulin and IGF-1, and a pro-inflammatory systemic environment, all of which are known to promote tumor growth and progression.

In contrast, dietary patterns rich in plant-based foods, whole grains, healthy fats, and fish, such as the Mediterranean diet or predominantly plant-based diets, are generally associated with improved overall survival and, in some cases, reduced risk of disease progression [61,63,65]. These beneficial effects may be explained by lower glycemic load, improved insulin sensitivity, reduced chronic inflammation, and a more favorable lipid profile. Additionally, such diets are rich in bioactive compounds, including polyphenols and antioxidants, which may further modulate oxidative stress and tumor biology.

Emerging evidence also suggests that dietary patterns may influence the tumor microenvironment through modulation of the gut microbiota, immune response, and systemic metabolic pathways. However, the heterogeneity of study designs, patient populations, and dietary assessment methods limits the ability to draw definitive conclusions regarding causality.

Taken together, these findings support the hypothesis that diet quality and metabolic health play a relevant role in PCa progression, although further well-designed prospective studies and interventional trials are needed to better define the impact of specific dietary strategies in this setting.

## 7. Ketogenic Diet and Low-Carbohydrate Diets in Preclinical Models of Prostate Cancer

Preclinical studies have provided valuable insights into how carbohydrate restriction and ketogenic dietary patterns may influence PCa progression (Table 1).

Early work by Freedland et al. showed that severe carbohydrate restriction can significantly slow PCa growth in vivo. In a xenograft model using SCID mice implanted with LAPC-4 human PCa cells, mice fed a no-carbohydrate ketogenic diet (NCKD—consisting of 84% fat, 0% carbohydrates, and 16% protein) had slower tumor growth compared to those fed a Western diet (WD—composed of 40% fat, 44% carbohydrates, and 16% protein), despite similar or higher calorie intake. This effect was partly explained by reductions in systemic insulin and modulation of the insulin-like growth factor (IGF) pathway suppressing the PI3K/AKT/mTOR signaling cascade [66].

Building on this, Mavropoulos et al. tested diets with varying carbohydrate and fat content in LNCaP xenograft-bearing mice: a very high-fat, no-carbohydrate ketogenic diet (NCKD: 83% fat, 0% carbohydrate, 17% protein), a low-fat, high-carbohydrate diet (LFD: 12% fat, 71% carbohydrate, 17% protein), or a high-fat, moderate-carbohydrate diet (MCD: 40% fat, 43% carbohydrate, 17% protein). The results showed that lowering carbohydrates while increasing fat intake decreased insulin and IGF-1 levels, prolonged survival and suppressed tumor proliferation [67].

In another study, Kim and colleagues investigated whether combining carbohydrate restriction with lactate transporter inhibition could enhance antitumor effects in a xenograft model of human PCa. Using mice implanted with human PCa cells, they tested a no-carbohydrate KD alongside an inhibitor targeting monocarboxylate transporters (MCTs), which mediate lactate export, a key step in cancer cell metabolic adaptation.

Their results showed that carbohydrate restriction alone reduced tumor growth and lowered insulin and IGF-1 levels, consistent with previous findings. Notably, combining the KD with lactate transporter inhibition further suppressed tumor growth compared to diet alone, suggesting a synergistic effect. This study supports the rationale for targeting both glucose metabolism and lactate export pathways as a strategy to disrupt PCa metabolism and slow disease progression [68].

In a preclinical study, Caso and colleagues tested whether dietary carbohydrate restriction could enhance tumor control in castrated mice with human PCa xenografts simulating the common clinical scenario of ADT. Mice fed a NCKD showed slower tumor growth and longer survival than controls on a standard Western diet and carbohydrate restriction led to lower insulin and IGF-1 levels, which are key mitogenic drivers in PCa progression. These findings support the hypothesis that limiting dietary carbohydrates may enhance the effects of castration by further suppressing metabolic pathways that fuel tumor growth, providing a rationale for combining carbohydrate restriction with standard ADT in PCa treatment [69].

In another study, Allott and colleagues examined how dietary carbohydrate restriction affects prostate tumor growth in obese conditions, using the Hi-Myc transgenic mouse model, which spontaneously develops PCa driven by c-Myc overexpression.

The results showed that carbohydrate restriction significantly reduced prostate tumor burden, lowered serum insulin, IGF-1, monocyte chemoattractant protein (MCP)-1 and interleukin (IL)-1α levels, reduced prostate macrophage infiltration and decreased body fat compared to obese mice fed the Western diet. These findings suggest that limiting dietary carbohydrates may mitigate the pro-tumor effects of obesity by reducing key metabolic and hormonal drivers of PCa growth and progression. However, this did not translate into a reduction in the incidence of adenocarcinoma [70].

Since realizing and maintaining an NCKD (0% carbohydrate kcal) in humans is quite impossible, Masko et al. in 2010 [22] conducted a study that explored the impact of different levels of dietary carbohydrate restriction (10–20% carbohydrate kcal) on PCa growth in mice, aiming to define how much carbohydrate reduction is needed to slow tumor progression effectively. The results highlighted that mice on a low-carbohydrate diet had similar survival as mice consuming NCKD.

Recent experimental evidence by Zhang et al. further expanded these findings by demonstrating that KD interventions can suppress PCa progression through multiple mechanisms. Their study revealed that glucose restriction via ketogenic feeding enhances oxidative stress in tumor cells and disrupts oncogenic pathways involved in growth and apoptosis, providing molecular evidence for a synergistic metabolic vulnerability [71].

Moreover, in murine models of metastatic PCa, the KD has been shown to reshape the epigenetic landscape, slow proliferation, and counteract oxidative stress. Specifically, it has been demonstrated that BHB acts as an endogenous inhibitor of class I histone deacetylases (HDAC2/3), promoting histone acetylation and the transcription of anti-inflammatory and antioxidant genes such as FOXO3a and metallothioneins. Regarding oxidative stress, it is reduced through activation of the Nrf2 pathway and decreased production of mitochondrial reactive oxygen species (ROS), potentially slowing tumor progression [74,76].

On the other hand, proteomic studies have revealed a significant upregulation of key ketogenesis enzymes (e.g., HMGCS2, ACAT1) in high-grade or castration-resistant prostate cancer (CRPC) [72,73,75,77]. These alterations suggest that more aggressive PCa cells may activate an alternative metabolic pathway that could be exploited as a therapeutic target.

Emerging evidence has also focused on the potential application of KD to enhance the efficacy of immunotherapies (anti-PD-1/CTLA-4) in resistant PCa. KD, increasing histone acetylation by acting as an endogenous inhibitor of class I histone deacetylases (HDAC2/3), enhanced the expression of genes involved in antigen presentation, such as MHC class I molecules. This effect promoted better recognition of tumor cells by cytotoxic T lymphocytes. In parallel, the ketogenic intervention reshaped the tumor immune landscape by increasing infiltration of CD8^+^ T cells, reprogramming tumor-associated macrophages towards a more pro-inflammatory M1 phenotype, and reducing immunosuppressive neutrophil infiltration [74].

Importantly, when combined with anti–PD-1 and anti–CTLA-4 antibodies, the KD amplified the response to ICB therapy, leading to significantly reduced tumor growth in otherwise resistant PCa models. These findings suggest that metabolic modulation through KD or BHB could represent a promising strategy to enhance the efficacy of immunotherapy in advanced or treatment-resistant PCa [74].

More recent preclinical studies, primarily based on murine models, have further strengthened this concept by demonstrating that KDs can profoundly reshape the epigenetic and immune landscape of prostate cancer. In particular, BHB has been shown to function as an endogenous histone deacetylase inhibitor, thereby promoting a more immunogenic tumor phenotype. Preclinical evidence indicates that KD may enhance the efficacy of immune checkpoint inhibitors by increasing CD8^+^ T-cell infiltration, improving antigen presentation, and reducing immunosuppressive cell populations within the tumor microenvironment. In parallel, emerging metabolic studies highlight the marked heterogeneity of PCa and its reliance on lipid metabolism, suggesting that dietary interventions targeting metabolic flexibility may represent a promising therapeutic strategy. These findings provide a more comprehensive and up-to-date mechanistic rationale for the integration of ketogenic approaches in PCa management [74,76,77].

Finally, the relationship between KD, systemic metabolism, and tumor biology can be conceptualized as an integrated metabolic axis. By significantly reducing dietary carbohydrate intake, KDs reduce circulating glucose levels, which in turn leads to decreased insulin secretion and reduced IGF-1 signaling. This downregulation of insulin/IGF-1 axis results in reduced activation of downstream oncogenic pathways, including PI3K/AKT/mTOR, which are central regulators of cell proliferation and survival. Furthermore, the metabolic environment induced by KDs imposes energetic constraints on tumor cells that rely heavily on glycolysis for ATP production and biosynthesis. While normal tissues can adapt by utilizing ketone bodies and fatty acid oxidation, many cancer cells exhibit limited metabolic flexibility, resulting in increased metabolic stress. This differential metabolic adaptation provides the biological rationale for the potential antitumor effects of KD interventions.

## 8. Ketogenic Diet and Low-Carbohydrate Diets in Clinical Trials

Although evidence from preclinical studies suggests promising antitumor effects of ketogenic and low-carbohydrate diets in PCa, clinical data remain limited but evolving.

One of the most notable human investigations is the CAPS2 trial, a 6-month, multicenter, randomized trial comparing a low-carbohydrate diet (LCD) to standard dietary habits in men with biochemically recurrent prostate cancer (BCR). The LCD group was instructed to limit carbohydrate intake to 20 g per day while the control group was advised to maintain their usual diet. A total of 27 participants in the LCD group and 18 in the control group completed the study. After six months the LCD group showed weight loss and metabolic benefits, in terms of improved insulin sensitivity, and significantly higher ketone levels, with a favorable trend toward longer PSA doubling time. In a post hoc exploratory analysis that adjusted for factors such as study PSA doubling time, baseline PSA levels, initial treatment type, and hemoconcentration effects, the LCD group showed a significantly longer PSA doubling time compared to the control group (28 months vs. 13 months; *p* = 0.021), suggesting potential slowing of biochemical disease progression [78].

In a secondary analysis, Chi et al. performed an in-depth serum metabolomic profiling of participants following a carbohydrate-restricted dietary intervention. The study found significant increases in circulating ketone bodies and alterations in lipid metabolites, indicating robust induction of nutritional ketosis. Importantly, higher ketone body levels were associated with slower PSA doubling time, suggesting that enhanced ketogenesis might contribute to decelerating tumor progression in this patient population. This metabolomic signature supports the mechanistic hypothesis that limiting glucose availability while boosting ketone metabolism could provide a metabolic disadvantage for prostate tumor cells [79].

In the same CAPS2 clinical trial cohort, Lin et al. in 2022 investigated the effects of LCD on intestinal permeability, measured by zonulin, a biomarker that reflects gut barrier function and is associated with inflammation and metabolic health, and systemic inflammation, assessed through high-sensitivity C-reactive protein (hsCRP) [80].

After six months, men with PCa who followed a LCD showed a significant reduction in serum zonulin levels, indicating improved gut barrier integrity. This effect was accompanied by modest weight loss and improved metabolic parameters. No changes were observed in hsCRP. These findings suggest that dietary carbohydrate restriction may have beneficial effects beyond weight control, potentially lowering systemic inflammation by enhancing intestinal barrier function in PCa patients. Moreover, linear regression indicated that greater weight loss was strongly associated with a longer PSADT (*p* = 0.003) [80].

Currently, direct comparisons between different therapeutic KD or low-carbohydrate dietary approaches in PCa are lacking. Most clinical studies have evaluated a single intervention, such as a low-carbohydrate diet or a specific ketogenic protocol, without directly comparing different levels of carbohydrate restriction, caloric intake, or macronutrient composition. As a result, it remains unclear whether more restrictive ketogenic approaches (e.g., classical or isocaloric ketogenic diets) offer additional benefits compared to less restrictive low-carbohydrate interventions. This gap in the literature limits the ability to define the optimal dietary strategy in terms of efficacy, safety, and long-term adherence. Future randomized trials directly comparing different dietary interventions are needed to establish the most effective and sustainable nutritional approach for patients with PCa.

## 9. Discussion

From a clinical safety perspective, several considerations should be addressed when implementing KDs or LCDs in patients with prostate cancer. A relevant concern is the increased risk of euglycemic ketoacidosis in patients treated with sodium-glucose cotransporter-2 (SGLT2) inhibitors, which are frequently prescribed in individuals with type 2 diabetes or metabolic syndrome. The combination of carbohydrate restriction and SGLT2 inhibition may further promote ketone body production, potentially increasing this risk and requiring careful clinical supervision [81,82,83].

In addition, potential metabolic interactions between KDs and ARPIs, such as enzalutamide, apalutamide, and darolutamide, should be considered. These agents are associated with metabolic alterations including weight gain, insulin resistance, dyslipidemia, and increased cardiovascular risk, which may overlap with or counteract the metabolic effects induced by KDs [84,85,86]. Although direct pharmacokinetic interactions are not well established, combined metabolic effects may influence patient tolerance and systemic metabolic homeostasis.

Therefore, careful clinical monitoring is essential during KD interventions. Recommended parameters include body weight and body composition, fasting glucose and ketone levels, lipid profile, renal and hepatic function, and markers of nutritional status, in line with current recommendations for metabolic monitoring in oncologic and metabolic interventions [35,87]. Multidisciplinary management involving oncologists, endocrinologists, and nutrition specialists is strongly advised to ensure safety and optimize therapeutic outcomes.

Another important consideration in the clinical application of KD in PCa is the dual role of their potential adverse effects. On one hand, metabolic changes induced by KD, such as weight loss, reduced insulin levels, and improved insulin sensitivity, may contribute to a less favorable environment for tumor growth. On the other hand, these same effects may be detrimental in cancer patients, particularly in those undergoing ADT, where loss of lean body mass, sarcopenia, and unintended weight loss are associated with worse clinical outcomes. Additionally, KDs may lead to alterations in lipid profiles and may be difficult to sustain over time, potentially limiting adherence. Importantly, the clinical impact of these effects may vary depending on patient characteristics. For instance, KDs or LCDs approaches may be more suitable in overweight or insulin-resistant patients, in whom metabolic improvements could provide additional benefit. In contrast, their use in frail patients or those at risk of malnutrition and sarcopenia should be approached with caution. Therefore, while KDs interventions may offer metabolic advantages, their implementation in patients with PCa requires careful patient selection, close nutritional monitoring, and individualized approaches to balance potential benefits and risks.

Based on available evidence, a pragmatic clinical framework can be proposed:

1. Patient selection: KD or LCD strategies may be most appropriate in patients with metabolic dysregulation, including obesity, insulin resistance, or features of metabolic syndrome, where reduction in insulin and IGF-1 signaling may provide additional biological benefit. Conversely, caution is advised in frail patients, those with sarcopenia, unintentional weight loss, or advanced disease-associated cachexia, particularly in the context of androgen deprivation therapy.
2. Clinical context: These interventions may be considered as supportive strategies in selected patients with localized disease under active surveillance or in those receiving systemic therapies (e.g., ADT or ARPI), with the aim of improving metabolic health and potentially modulating treatment-related adverse effects. At present, there is insufficient evidence to recommend ketogenic diets as anti-tumor therapy per se.
3. Monitoring and safety: Patients undergoing dietary intervention should be closely monitored for body weight, body composition, fasting glucose, lipid profile, renal and hepatic function, and ketone levels. Special attention should be given to the risk of sarcopenia, dyslipidemia, and treatment-related metabolic interactions, particularly in patients receiving SGLT2 inhibitors or androgen receptor pathway inhibitors.
4. Implementation framework: Dietary interventions should be individualized, time-limited, and supervised. Integration with structured nutritional counseling and physical activity programs is essential to preserve lean body mass and optimize metabolic outcomes.

Overall, KD and LCD dietary strategies should be viewed as part of a broader concept of precision nutrition in oncology, rather than as uniform interventions applicable to all patients with prostate cancer.

## 10. Strengths and Limitations

This review provides a comprehensive and up-to-date synthesis of the available evidence on the role of the KD in PCa, integrating data from preclinical models, clinical studies, and mechanistic research. A major strength lies in the multidimensional approach, which considers metabolic, endocrine, and oncologic perspectives, as well as the interaction between dietary interventions and standard hormonal therapies. In addition, the inclusion of both experimental and early clinical data allows for a broad overview of potential biological mechanisms and translational implications. However, several limitations should be acknowledged. The available clinical evidence is still limited, with small sample sizes, short follow-up durations, and heterogeneity in study design, patient populations, and KD protocols, which restricts the generalizability of the findings. Moreover, most data derive from preclinical studies, and their applicability to human disease remains uncertain. Variability in dietary adherence and the potential risk of confounding factors further complicate interpretation. Finally, the lack of robust randomized controlled trials with hard oncologic endpoints prevents definitive conclusions regarding the efficacy and safety of ketogenic interventions in prostate cancer.

## 11. Conclusions

**Figure 1:**
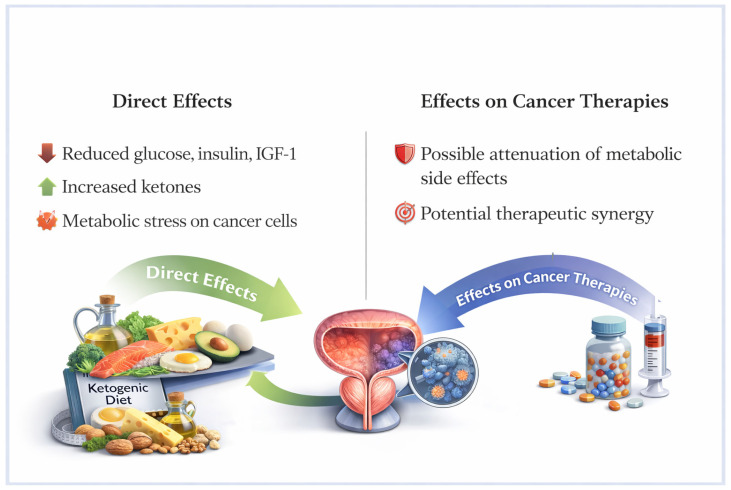
Potential impact of ketogenic diet on prostate cancer.

The available studies suggest that metabolic modulation represents a biologically plausible strategy to influence PCa progression, particularly in the context of AR-driven metabolic reprogramming (Figure 1). The KD appears to target key metabolic pathways involved in tumor growth, such as insulin/IGF-1 signaling, and glucose and lipid metabolism, and may partially mitigate the adverse metabolic effects induced by ADT and ARPIs. Preclinical studies consistently support these mechanisms, demonstrating reduced tumor growth, modulation of oncogenic signaling pathways, and potential synergistic effects with standard treatments, including hormonal therapy and immunotherapy. However, clinical evidence remains limited and heterogeneous, with preliminary findings suggesting improvements in metabolic parameters and possible delays in biochemical progression, but without definitive confirmation on clinically relevant oncologic outcomes. Importantly, variability in KD protocols, patient selection, and adherence represents a significant challenge in translating these approaches into routine practice. Therefore, while the KD may represent a promising adjunctive strategy within a multimodal treatment framework, its use should currently be considered investigational and restricted to controlled settings or under strict clinical supervision. Future research should focus on well-designed randomized controlled trials to clarify patient selection, optimal dietary protocols, long-term safety, and its impact on survival and disease progression.

## Figures and Tables

**Table 1:** Summary of preclinical studies investigating ketogenic and low-carbohydrate diets in prostate cancer models.

Study	Experimental Module	Model	Dietary Intervention	Outcomes	Principal Findings
Freedland, 2008 [66]	xenograft murine model	SCID mice implanted with LAPC-4 human cells	NCKD vs. Western diet	tumor growth, insulin levels, IGF pathway/PI3K-AKT-mTOR signaling	NCKD reduced tumor growth; associated with reduced insulin levels and modulation of the IGF pathway/PI3K-AKT-mTOR signaling
Mavropoulos, 2009 [67]	xenograft murine model	LNCaP xenograft-bearing mice	NCKD vs. LFD and MCD	tumor proliferation, survival, insulin and IGF-1 levels	NCKD decreased insulin and IGF-1 levels, prolonged survival and suppressed tumor proliferation
Masko, 2010 [22]	xenograft murine model	SCID mice implanted with LAPC-4 human cells	LCD vs. NCKD	tumor growth, survival	LCD achieved similar survival to NCKD
Kim, 2012 [68]	xenograft murine model	mice implanted with human PCa cells	NCKD combined with MCT inhibitor vs. NCKD alone	tumor growth, insulin and IGF-1 levels	NCKD alone reduced tumor growth and lowered insulin and IGF-1 levels; combination with MCT suggested a synergistic effect
Caso, 2013 [69]	xenograft ADT murine model	mice implanted with human PCa cells under castration conditions	NCKD vs. WD	tumor growth, survival, insulin and IGF-1 levels	NCKD reduced tumor growth and improved survival under castration, associated with lower insulin/IGF-1
Allott, 2017 [70]	transgenic mouse model	Hi-Myc transgenic mice under obese conditions	carbohydrate-restricted diet vs. WD	tumor burden, metabolic and inflammatory markers (insulin, IGF-1, MCP-1, IL-1α), macrophage infiltration, body fat, adenocarcinoma incidence	carbohydrate restriction reduced tumor burden and metabolic/inflammatory markers under, without effect on adenocarcinoma incidence
Zhang, 2018 [71]	cellular model	PL-3 cell line	N.A.	ketolytic enzyme levels (BDH1 and OXCT1)	PCL-3 cells showed moderate expression of ketolytic enzymes, suggesting a possible response to the KD
Saraon, 2013, 2014 [72,73]	preclinical molecular/proteomic study	castration-resistant prostate cancer models (CRPC); human PCa tissue	N.A.	Expression of ketogenesis-related enzymes (HMGCS2, ACAT1, BDH1, HMGL, OXCT1)	ketogenesis enzymes (HMGCS2, ACAT1) upregulated in high grade/metastatic CRPC
Murphy, 2024 [74]	xenograft murine model of metastatic prostate cancer	mice implanted with various PCa cells	KD alone or in combination with immune checkpoint inhibitors (anti-PD-1/CTLA-4) vs. SD ± immunotherapy	tumor proliferation, oxidative stress, epigenetic modulation (HDAC activity, histone acetylation), gene expression (FOXO3a, metallothioneins; tumor immune microenvironment, antigen presentation (MHC-I), CD8^+^ T cell infiltration, macrophage polarization, neutrophil infiltration	- KD reduced proliferation and oxidative stress via epigenetic modulation (BHB-mediated HDAC inhibition and increased antioxidant gene expression) - KD enhanced antitumor immunity (increased HC-I, CD8^+^ T cells and M1 polarization, reduced neutrophils)
Yum, 2025 [75]	xenograft CRPC murine model	castration-resistant C4-2 xenograft mouse model	ketone supplementation vs. SD	Tumor growth, epigenetic regulation (EZH2 activity)	Ketone supplementation inhibited tumor growth via EZH2 targeting and epigenetic modulation

## Data Availability

The original contributions presented in this study are included in the article. Further inquiries can be directed to the corresponding authors.

## Abbreviations

The following abbreviations are used in this manuscript:

NCKD	no-carbohydrate ketogenic diet
LFD	Low-fat diet
MCD	moderate-carbohydrate diet
MCT	monocarboxylate transporter
ADT	androgen deprivation therapy
LCD	Low-carbohydrate diets
KD	ketogenic diet
SD	standard diet
WD	Western diet
CRPC	castration-resistant prostate cancer
BHB	β-hydroxybutyrate
